# On-admission serum uric acid predicts outcomes after acute myocardial infarction

**DOI:** 10.3325/cmj.2012.53.642

**Published:** 2012-12

**Authors:** Chunkui Zhou, Jiang Wu, Shaokuan Fang

**Affiliations:** Department of Neurology, the First Teaching Hospital of Jilin University, Changchun, China

**To the Editor:** In a previous issue of the *Croatian Medical Journal*, Trkulja and Car (1) reported that higher on-admission serum uric acid (SUA) independently predicted worse short-term and medium/long-term outcomes after acute myocardial infarction (AMI). Although the data were analyzed by statistical methods, the conclusion should be interpreted with caution.

Some studied have suggested that serum uric acid predicts coronary heart disease (CHD) (2). However, prospective epidemiological studies have reported apparently conﬂicting ﬁndings, with several studies reporting positive associations only among women (3,4). Wheeler et al (5) reported that serum uric acid levels were unlikely to predict CHD, and this factor was unlikely to be a major determinant of the disease in the general population. Uric acid is an “antioxidant,” a free radical scavenger, and a chelator of transitional metal ions, which are converted to poorly reactive forms (6). Temporary hyperuricemia may afford the beneficial antioxidant effects of urate (7). Depending on its level, serum urate may exhibit protective and deleterious effects on stroke outcomes. More patients with low (<280 μM) and high (>410 μM) urate levels had poor functional outcomes (36% and 27%, respectively), compared to those with urate levels between 340 and 410 μM (14%) (8).

Furthermore, limitations of this meta-analysis may arise from the inevitably nonrandom choice of independent studies. Also, the same standard was used for studies with obvious disparities. Therefore, a more specific study on this complicated clinical problem is required.

In our opinion, serum uric acid levels need to be stratified to determine the definite relationship between serum uric acid level and the outcomes after acute myocardial infarction.
